# Insomnia treatment based on the regulation of the GABAergic system: traditional Chinese medicine perspectives and therapeutic approaches

**DOI:** 10.3389/fnins.2025.1670273

**Published:** 2026-01-12

**Authors:** Chen-zhe Cheng, Ming-zhen Xie, Yuan Tian, Wen-qi Qiao, Ze-yu Yu, Shi-qing Wang, Feng Yun

**Affiliations:** 1 Heilongjiang University of Traditional Chinese Medicine, Harbin, Heilongjiang, China; 2 The Third Affiliated Hospital of Heilongjiang University of Traditional Chinese Medicine, Harbin, Heilongjiang, China

**Keywords:** traditional Chinese medicine, GABAergic system, insomnia, Chinese herbal formulas, Chinese patent medicine, herbal

## Abstract

Insomnia is a common clinical manifestation of central nervous system (CNS) dysfunction that may be attributable to the involvement of abnormalities in multiple systems. Dysfunction of the gamma-aminobutyric acid (GABA) system is one of the key pathologic factors responsible for the increased neural excitability. GABA has been regarded as the major inhibitory neurotransmitter in the CNS, which can maintain the dynamic balance of neural networks by regulating neuronal excitability. Traditional Chinese medicine (TCM), with its multi-component, multi-target therapeutic advantages, shows unique potential in regulating the GABAergic system to improve sleep disorders. Accordingly, this review systematically elucidated the underlying mechanisms of TCM in treating insomnia by regulating the GABAergic system. According to the results, the intervention pathways of TCM exhibited multidimensional characteristics. TCM could effectively enhance GABA levels, strengthen GABAergic neural signal transmission, and improve insomnia symptoms by activating GABA receptor expression, upregulating glutamic acid decarboxylase (GAD) expression, and inhibiting GABA transaminase (GABA-T) activity. Furthermore, TCM could regulate the function of the hypothalamic–pituitary–adrenal (HPA) axis, inhibit neuroinflammation, regulate circadian rhythms, and enhance neurotrophic mechanisms, thereby synergizing with the GABAergic system to exert a sedative and sleep-promoting effect. With a systematic elaboration of the mechanisms of the GABAergic system, findings in this study may provide a theoretical basis for establishing a TCM evaluation system for insomnia based on the modulation of the GABAergic system. It can also offer fresh insights into the diagnosis and treatment of insomnia from the perspective of TCM, expand new directions for clinical research, and supply potential reference for future in-depth studies.

## 1 Introduction

Insomnia, also known as sleeplessness, is a common sleep disorder and mental illness clinically (Benca and Buysse, 2018). Patients with insomnia may usually experience difficulty falling asleep or maintaining sleep, accompanied by irritability and fatigue during waking hours. Primary insomnia refers to its independent presence as a mental illness. Secondary insomnia, on the other hand, often coexists with other underlying diseases, mental disorders, or iatrogenic factors as a concomitant symptom (Buysse, 2013; Fornaro et al., 2024). According to the first meta-analysis on the prevalence of insomnia among the general Chinese population conducted by Cao et al. (Cao et al., 2017) the prevalence rate reached 15%; and the combined prevalence was significantly lower in individuals with an average age ≥43.7 years compared to those aged <43.7 years, suggesting a trend toward younger onset of insomnia in China. Meanwhile, the America Insomnia Survey indicates that approximately 23.2% of the working population suffers from insomnia, and its incidence is on the rise in the United States (Dopheide, 2020). Insufficient sleep has been proven to be a risk factor for various diseases, such as hypertension (Fernandez-Mendoza et al., 2012), diabetes (Vgontzas et al., 2009), cardiovascular and cerebrovascular diseases (Nambiema et al., 2023; Gottesman et al., 2024), cancer (Shi et al., 2020), depression (Li et al., 2016), and anxiety disorders (Chellappa and Aeschbach, 2022), and also as a complication of several diseases such as respiratory system diseases (Bjornsdottir et al., 2020) and mental disorders (Hertenstein et al., 2022). For mental disorders (e.g., depression and anxiety), insomnia serves both as a risk factor and a secondary symptom, which is prone to form a vicious cycle in the affected individuals (Mirchandaney et al., 2022). Given the prevalence of insomnia and its impact on patients’ subjective well-being and physiological homeostasis, it is pivotal to treat and prevent insomnia effectively, thereby protecting the physical and mental health of the population. Insomnia, referred to as “bu mei” in traditional Chinese medicine (TCM), is characterized by the inability to fall asleep normally. TCM is an assortment of traditional medical practices that originated thousands of years ago, and has garnered widespread attention for its unique philosophy and therapeutic approaches. Long-term use of alprazolam, the most commonly prescribed hypnotic medication in China, may lead to adverse effects such as dependence, cognitive decline, and psychiatric issues. This has prompted an increasing number of patients with insomnia to seek alternative therapies. Among these, TCM has been recognized to be one of the mature and comprehensive traditional medical systems that is gaining greater attention (Singh and Zhao, 2017). In the management of insomnia, TCM can offer advantages such as low drug dependency and personalized treatment (Tang et al., 2021). Conventionally, TCM focuses on macro-level regulation of the body, but over time, understanding of the roles of TCM in disease treatment has evolved from a macro to a micro perspective (Li et al., 2025). In recent years, TCM has been increasingly recognized for its unique position in the field of insomnia (Li et al., 2020; Zhang et al., 2019).

**Figure 1:**
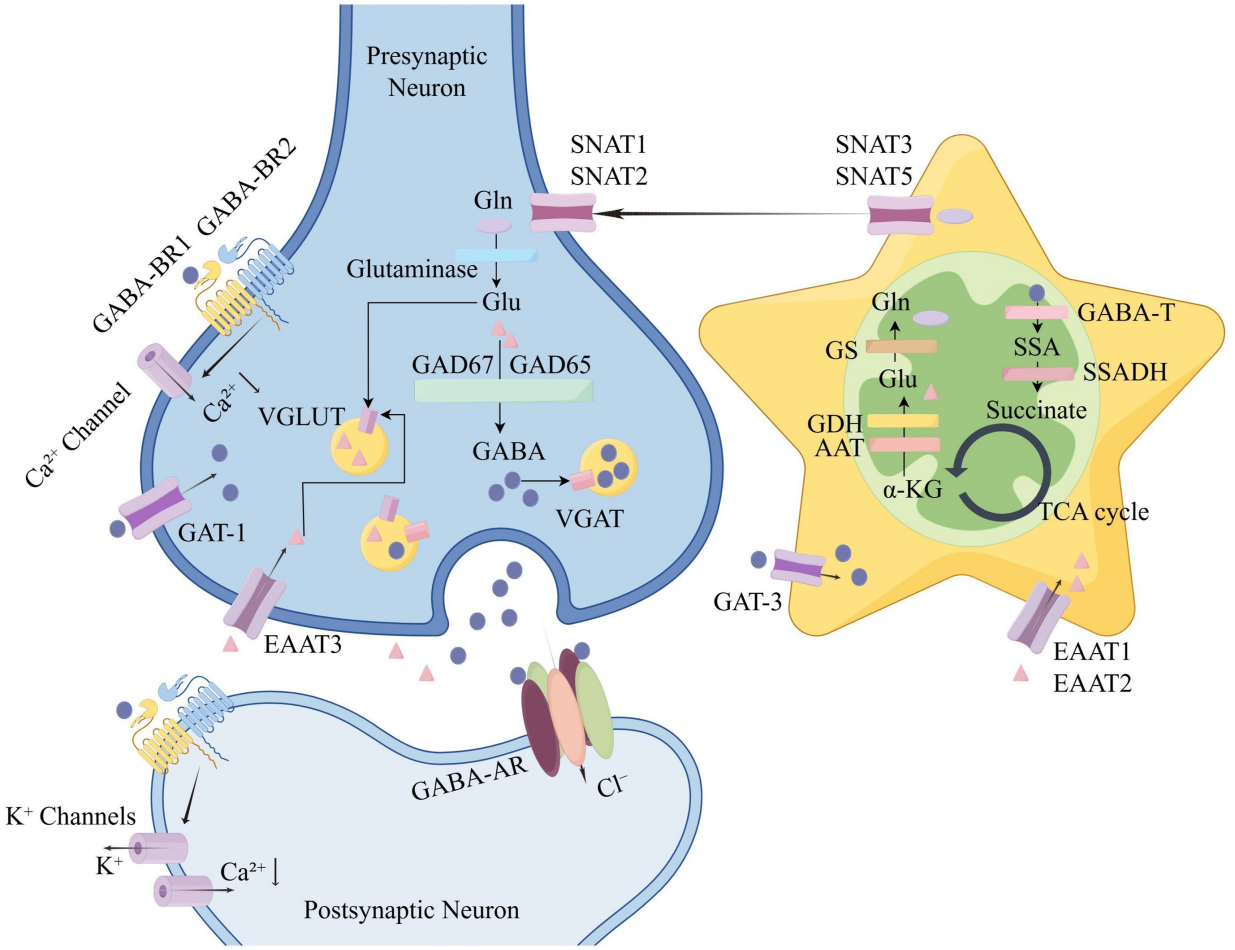
Diagram of the mechanism of the GABAergic system. Diagram of the mechanism of the GABAergic system using Figdraw (www.figdraw.com). Glu is produced by the hydrolysis of Gln catalyzed by glutaminase. Glu may undergo decarboxylation to form GABA, owing to the effect of GAD (Zhang X. H. et al., 2023; Sears and Hewett, 2021; Andersen, 2025). After metabolism by GABA-T, GABA is transformed into SSA, followed by the conversion into succinate due to the action of SSADH, thereby entering the TCA cycle (Kabała and Janicka, 2024). In the Glu/GABA-Gln cycle, Gln is almost entirely synthesized within astrocytes. Glu can be synthesized from *α*-KG, a product of the TCA cycle, via AAT, GDH, then catalyzed by GS to form Gln, eventually being transported into neurons via SNAT1/SNAT2 after releasing into the synaptic cleft via glutamine transporters SNAT3/SNAT5 (Andersen, 2025; Tecson et al., 2025; Mamelak, 2024; Bolneo et al., 2022). Gln, glutamine; Glu, glutamate; GAD65/67, glutamic acid decarboxylase 65/67-kDa isoform; GABA, gamma-aminobutyric acid; VGAT, vesicular GABA transporter; VGLUT, vesicular glutamate transporter; GAT-1/3, gamma-aminobutyric acid transporter 1/3; EAAT1-3, excitatory amino acid transporter 1–3; SNAT1/2/3/5, sodium-neutral amino acid transporter1/2/3/5; GABAR, GABA receptors; GABA-T, GABA transaminase; SSA, succinic semialdehyde; SSADH, succinic semialdehyde dehydrogenase; TCA cycle, tricarboxylic acid cycle; α-KG, α-ketoglutarate; AAT, aspartate aminotransferase; GDH, glutamate dehydrogenase; and GS, glutamine synthetase.

The gamma-aminobutyric acid (GABA) system has been proven to possess pivotal functions such as maintaining the neural circuit balance through the regulation of the neuronal excitability within the central nervous system (CNS) given its primary role as an inhibitory neurotransmitter (Roth and Draguhn, 2012). GABA, as a neurotransmitter, is widely distributed throughout the mammalian CNS and serves as the primary medium for synaptic inhibition (Miller, 2019). Glutamic acid (Glu) is produced through the hydrolysis of glutamine (Gln) catalyzed by glutaminase, which participate in maintaining neural excitability as a precursor to GABA; meanwhile, glutamic acid decarboxylase (GAD) is the key enzyme converting Glu into GABA (Zhang X. H. et al., 2023; Sears and Hewett, 2021; Andersen, 2025), GABA transaminase (GABA-T) is the key enzyme in GABA metabolism (Kabała and Janicka, 2024). Both GAD and GABA-T play crucial roles in regulating GABA concentration, and GABA can also be recycled back into Gln (Figure 1) (Andersen, 2025; Tecson et al., 2025; Mamelak, 2024; Bolneo et al., 2022). The termination of GABAergic signaling depends primarily on the reuptake of GABA transporter (GAT) on neurons and glial cells (Nayak et al., 2023). GABAergic neurons show extensive distribution across a variety of brain regions, such as the cerebral cortex, striatum, hippocampus, globus pallidus, amygdala, hypothalamus, etc. Serving as a promising drug target, GABA functions crucially in the treatment of insomnia, diabetes, memory loss, depression, and other neurological or metabolic disorders (Jiang, 2021). GABA has been revealed to possess a neuroprotective effect in insomnia through mechanisms such as inhibiting neuroinflammation and repairing oxidative damage (Zhu et al., 2024). Furthermore, the sleep-promoting role of GABA relies on its binding to GABA receptors (GABARs). The activation of GABA_A_ receptor (GABA_A_R) is involved in the onset of sleep, and a decrease in their expression may in turn disrupt sleep homeostasis. mRNA expression of the GABA_A_R α1/α2 subunits in peripheral blood has been measured to be significantly reduced in insomniac patients (Xiang et al., 2023). Conversely, partial positive allosteric modulators of GABA_A_R can increase total duration of sleep and improve sleep efficiency in insomniac patients, while reducing wake after sleep onset and latency to sustained sleep (Huang et al., 2024). A previous animal study indicated that GABA_B_R agonists decreased rapid eye movement (REM) sleep duration, increased non-REM sleep duration, and reduced sleep latency in rats (Wisor et al., 2006); and another study found that GABA_B_R antagonists (CGP 35348) diminished slow-wave sleep in rats (Gauthier et al., 1997). In summary, impaired function of the GABAergic system is an important underlying factor in the development of insomnia.

This paper explores and analyzes the direct and indirect mechanisms by which the GABAergic system influences sleep disorders, combined with systematic elucidation of the regulatory functions of TCM on this system, and discussion of the development prospects and advantages of using TCM to treat insomnia through the GABAergic system, with an aim of offering fresh treatment strategies and methods for relieving sleep disorders. Literature review was realized based on the identification of relevant articles included in online public databases (e.g., PubMed, Web of Science, Embase, CNKI, etc.), with keywords including insomnia, sleep disorders, and GABA.

## 2 GABAergic system and insomnia

Currently in the clinical setting, there have been several methods targeting the GABAergic system available for treating insomnia, such as the activation and modulation of GABA receptors. Other approaches, including modulation of GABA synthesis and degradation, inhibition of GABA reuptake, and regulation of vesicular transporter function, remain in animal studies with the requirement for validating corresponding feasibility (Bolneo et al., 2022; Walsh et al., 2009; Walzer et al., 2021; Matsunaga et al., 2023; Si W. et al., 2020; Chaturvedi et al., 2022). Figure 1 illustrates the processes of GABA synthesis and metabolism. Existing evidence unveils that some of the aforementioned methods may also exert sedative and hypnotic effects synergistically and indirectly through mechanisms such as suppressing neuroinflammation, repairing oxidative damage, enhancing neurotrophic support, regulating circadian rhythms, and stabilizing the hypothalamic–pituitary–adrenal (HPA) axis (Zhu et al., 2024; Arenas et al., 2022; Patton et al., 2023; Giordano et al., 2006).

### 2.1 Direct regulation of the GABAergic system for the treatment of insomnia

#### 2.1.1 Activation and regulation of GABA_A_R

GABA_A_R has been documented to be the primary mediator of rapid inhibitory neurotransmission within the brain. By binding to it, GABA can promote the opening of the intrinsic chloride (Cl^−^) channels, and increase of the Cl^−^ conductance to pose challenge for neurons to generate action potentials. Current investigation has identified 19 human GABA_A_R subunits, including α1-6, β1-3, γ1-3, δ, ε, θ, π, and ρ1-3 (Sun et al., 2023). The most common GABA_A_R is composed of five subunits arranged in a counterclockwise direction as β-α-β-α-γ, forming a pentameric ligand-gated Cl^−^ channel around a central axis (Kim and Hibbs, 2021). Three main structural domains are contained in each subunit, namely, the extracellular domain (ECD), the transmembrane domain (TMD), and the intracellular domain (ICD). The ECD forms the GABA binding site at the β+/α − subunit interface and the high-affinity binding site for benzodiazepine (BZD) at the α+/γ − subunit interface, while the TMD forms and regulates the Cl^−^ channel pore. Among these, the β+/α − interface acts as a low-affinity binding site, which is the major target for anesthetics and sedatives (e.g., etomidate, and propofol). BZD such as diazepam can also interact with this site, and drug binding here can exert a direct regulatory effect on the gating state of the ion channel (Iorio et al., 2020). The GABA_A_-*ρ* receptor (GABA_A_-ρR) is a subtype of the GABA_A_R, formed by the ρ1, ρ2, and ρ3 subunits through homotypic or pseudohomotypic oligomerization into a pentameric structure. It can be found frequently within the upper thalamus, cerebellum, and hippocampus. Among these, *ρ*1 exhibits an obvious expression in the retinal region (Gavande et al., 2010). GABA_A_-ρ1R, despite poorly understood physiological functions, has been uncovered to have a pivotal role in sleep–wake behavior in rats, and the selective distribution of ρ subunits suggests the research potential of GABA_A_-ρR in visual image processing (Naffaa et al., 2017). As demonstrated by a crossover study, a single dose of 1.5 mg or 2.5 mg of EVT 201, a partial positive allosteric modulator of benzodiazepine GABA_A_RS, could significantly improve pathological indicators in patients with primary insomnia. These improvements manifested as increased total sleep time, reduced frequency of awakenings, shortened time to sleep onset, and decreased sleep latency (Walsh et al., 2009). To sum up, activation of GABA_A_R can reduce neural excitation and improve sleep disorders.

#### 2.1.2 Activation and regulation of GABABR

GABA exerts its role as the primary inhibitory neurotransmitter in the brain through both ionotropic and metabotropic receptors. Unlike the source of GABA_A_R from the ionotropic receptor family, the metabotropic GABA_B_R is a G protein-coupled receptor found on both excitatory and inhibitory synapses in nearly all brain regions (Chalifoux and Carter, 2011). With two subunits (GABA_B1_ and GABA_B2_), GABA_B_R functions as a heterodimer. Each subunit contains a large ECD (vertical filament domain, VFT), a seven-TMD, and an intracellular C-terminal domain (Xu et al., 2014). Specifically, GABA_B1_ is responsible for binding GABA, while GABA_B2_ for coupling G proteins and enhancing the affinity of GABA for GABA_B1_. GABA_B1_ has two variants, GABA_B1a_ and GABA_B1b_, which differ in that the N-terminal ECD of GABA_B1a_ contains a pair of sushi repeat domains. The heterodimeric GABA_B1_/GABA_B2_ complex is the minimal functional unit required for receptor signaling, among which GABA_B1a_/GABA_B2_ and GABA_B1b_/GABA_B2_ are enriched at the axon terminal and in the soma-dendritic region, respectively. Meanwhile, serving as axonal transport signals, the sushi domains can stabilize the GABA_B1a_/GABA_B2_ receptor on the cell surface (Fritzius and Bettler, 2020; Huang, 2006). GABA_B_R is present in both presynaptic and postsynaptic regions. Presynaptic GABA_B_R can inhibit neurotransmitter release by inhibiting voltage-gated calcium channels (CaV); while postsynaptic GABA_B_R, when activated, is primarily responsible for inwardly opening rectifying potassium channels, generating slow inhibitory post-synaptic potential (IPSP), and also inhibiting CaV (Kantamneni, 2015; Jurčić et al., 2019). Existing clinical data has documented that the use of ASP8062, a positive allosteric modulator of GABA_B_RS, at a single dose of 35 mg or 70 mg could dose-dependently prolong the duration of overnight slow-wave sleep in patients with primary insomnia significantly. This effect was primarily concentrated in the first third of the night, without effect on the duration of REM sleep (Walzer et al., 2021). Therefore, the activation of GABA_B_R also exerts an inhibitory effect on neural excitation to play a sleep-promoting role.

#### 2.1.3 Promoting GABA synthesis

GAD, with wide expression in the CNS, is a rate-limiting enzyme that is responsible for synthesizing GABA (Dalakas, 2022). GAD65 and GAD67 are two GAD enzyme subtypes distributed in mammals (Wang J. et al., 2024), of which the former one is mainly enriched in axon terminals, while the latter one has a wide distribution throughout neurons (Kaufman et al., 1991). GAD65 and GAD67 are Class II pyridoxal 5′-phosphate (PLP)-dependent fold type I enzymes. The interaction between apoGAD and PLP is the key to regulate the activity of GAD in the short term. GAD67, remaining in an active state usually, is responsible for the basal production of GABA; while at least 50% of GAD65 exists in an inactive apoenzyme form, but it can be activated when additional GABA synthesis is required (Kass et al., 2014). GAD65 can rapidly produce GABA for release in an activity-dependent manner, while GAD67 is localized within the cytoplasm and synthesizes GABA for the metabolic needs of cells. In the absence of GAD65, GAD67 can, to some extent, produce GABA for vesicle release (Müller et al., 2015). In a prior experiment on gene-knockout mice (Matsunaga et al., 2023), GAD67 was expressed during early development, while GAD65 developed during postnatal maturation. GAD65 gene-knockout mice can survive into adulthood, but their brain GABA concentrations are only 50–75% of those in wild-type mice, and they exhibit epilepsy susceptibility and abnormal emotional behaviors. In the context of blocked GAD65 enzyme activity, there may be disrupted synthesis of GABA, and impaired function of inhibitory GABAergic neural circuits, thus forming a state of excessive excitation in the CNS ultimately (Muñoz-Lopetegi et al., 2020). Further investigation within this field also indicated long-term reductions in GAD67 levels and GABA concentrations in GAD67 haploinsufficiency mice, leading to reduced axonal branching, decreased numbers of GABAergic interneurons, and impaired cellular maturation, thereby causing disrupted GABAergic inhibitory circuits (Bolneo et al., 2022). As proven clinically, when patients exhibited extremely high titers of GAD65 autoantibodies in both serum and cerebrospinal fluid, there would be an emergence of a triad of symptoms, manifesting as severe sleep disturbances characterized by daytime somnolence and nocturnal insomnia (unresponsive to conventional treatments), combined with axonal sensory-motor polyneuropathy and autonomic neuropathy. This triad was proposed to constitute a novel phenotype associated with GAD65 autoimmunity (Kesserwani, 2020). Altogether, defects in either GAD65 or GAD67 subunit can produce severe dysfunction of the GABAergic neural system, disrupting the excitatory-inhibitory balance of the neural network, and ultimately leading to excessive excitatory states in the CNS and associated behavioral abnormalities. GAD65 autoimmunity is associated with severe sleep disorders unresponsive to conventional treatment.

#### 2.1.4 Inhibition of GABA degradation

GABA-T is a core regulatory molecule maintaining dynamic neuronal excitation-inhibition balance in the CNS. As a key catalytic enzyme in GABA degradation metabolism, GABA-T, with suppressed activity, can block the degradation process of GABA, significantly increasing GABA concentration, and thereby maintaining neurotransmitter homeostasis by reducing neurotransmitter degradation (Li et al., 2022). In rats with metabolic syndrome, elevated GABA-T activity in the cerebral cortex and hippocampus would reduce GABA levels and function indirectly, leading to increased anxiety-like behavior, prolonged sleep latency, and impaired spatial memory (Srichomphu et al., 2022). Sleep disorders can negatively impact the seizure threshold and frequency of epileptic seizures, while epileptic seizures can also affect sleep quality (Reddy et al., 2018). Vigabatrin, as an anticonvulsant drug, is an irreversible inhibitor of GABA-T, which can work to elevate the level of GABA in brains of rats, mice, and humans, underlining a direct role of GABA-T in GABA metabolism within the CNS (Si W. et al., 2020). These findings also highlight the potential application value of GABA-T in the treatment of neuropsychiatric disorders, beyond the confirmation of its important role in GABA catabolism. Under specific pathological conditions, reducing GABA-T activity can effectively increase GABA levels in the brain, thereby enhancing GABAergic inhibitory function and improving symptoms such as anxiety and sleep disorders caused by imbalances in these neurotransmitters.

#### 2.1.5 Inhibition of GABA reuptake

GABA levels are subjected to regulation by the activity of GATs on presynaptic and glial cell membranes. Multiple GAT subtypes mediate Na^+^/Cl^−^-dependent GABA uptake in neurons, glial cells, etc. (Joseph et al., 2022). GATs can uptake GABA from the synaptic cleft, with GAT-1 and GAT-3 being the most prominently expressed (Keros and Hablitz, 2005). GAT-1 and GAT-3 belong to the neurotransmitter sodium symporter family and follow the classic “LeuT” folding pattern, consisting of 12 transmembrane helices (Yadav et al., 2025), GAT-1 and GAT-3 are accompanied by 2 Na^+^ and 1 Cl^−^ ions during the transportation of a single GABA molecule, resulting in an electrically driven transmembrane influx of GABA. Through this process, GABA is removed from the synaptic cleft, resulting in the termination of GABA-mediated postsynaptic inhibitory currents (IPSCs) (Joseph et al., 2022; Ueda and Willmore, 2000). As the most highly expressed GAT in cortical structures, GAT-1 is dominantly responsible for GABA uptake by neurons. GAT-3 is another highly expressed cortical GAT that is mainly distributed in glial cells (Savtchenko et al., 2015). Increased GAT-3 activity can lead to the accumulation of Na^+^ in astrocytes, thereby increasing Ca^2+^ levels within astrocytes through Na^+^/Ca^2+^ exchange, resulting in the release of ATP/adenosine from astrocytes. Subsequently, the elevated extracellular adenosine levels can then act on presynaptic A1 adenosine receptors. This signaling pathway synergizes with the previously proposed GABA_B_R-mediated inhibitory mechanism of excitatory transmission (Boddum et al., 2016). In another study (Chaturvedi et al., 2022), pharmacological inhibition of GAT-1 increased non-REM sleep and shortened the latency period of non-REM sleep. Mechanistically, the reduced sleep may be explained by the reason that neuropathic pain can activate astrocytes and increase the expression of GAT-3 in astrocytes in the anterior cingulate cortex. Collectively, the termination of GABAergic signaling primarily depends on the activity of GAT-1 and GAT-3, which maintain neural excitability balance by clearing GABA from the synaptic cleft, regulate GAT levels, and inhibit GABA reuptake, thereby serving as one of the mechanisms for improving sleep disorders.

#### 2.1.6 Regulation of vesicle transporter function

In most cases, Glu mediates the excitatory synaptic transmission, and GABA modulates the inhibitory synaptic transmission, through vesicular Glu transporters 1–3 (VGLUT1–3) and vesicular GABA transporter (VGAT), respectively, and are taken up and accumulated in synaptic vesicles. VGLUT1-3 and VGAT are co-expressed, and synapses co-expressing VGLUT1 and VGAT may participate in the regulation of the excitatory-inhibitory balance in cortical microcircuits through activity-dependent changes in their numbers (Fattorini et al., 2009; Fattorini et al., 2015). Through fusion with the plasma membrane and release Glu, Glu-loaded vesicles may further trigger the activation of ionotropic receptors (iGluRs) including NMDAR (NR1, NR2, and NR3 subunits), AMPAR (a tetramer composed of GluR1-4 subunits), KAR, and metabotropic receptors (mgluRs). Ion channel-type receptors can mediate rapid signal transmission by coupling to ion channels; metabotropic receptors, also exert slow physiological effects through G protein coupling. Subsequently, excitatory amino acid transporter (EAAT) 3 reupsects Glu from the synaptic cleft for reloading into vesicles for recycling, while EAAT2 (also known as Glu transporter 1, GLT-1) and EAAT1 are expressed in astrocytes to prevent excessive accumulation of Glu in the synaptic cleft (Keros and Hablitz, 2005; Yadav et al., 2025; Parkin et al., 2018; Zhou and Danbolt, 2013; Qiao et al., 2022; Thompson and Chao, 2020). Via VGAT, GABA is loaded into synaptic vesicles, and by vesicular exocytosis, it is then released into the synaptic cleft, which may further bind to receptors and trigger a series of reactions subsequently (Juge et al., 2013). In a previous experiment, VGAT-deficient mice were observed with nearly absent spontaneous GABAergic IPSCs within the spinal motor, hypoglossal motor and cultured neurons, directly demonstrating the role of VGAT deficiency in causing severe defects during functional GABAergic synaptic transmission (Bolneo et al., 2022). Further evidence from mouse VGAT gene knockout research confirms that VGAT is the sole transporter for GABA inhibitory neurotransmitters, and VGAT is co-expressed with GAD65, GAD67, and GAT-1 (Münster-Wandowski et al., 2016). In summary, there is a co-expression of VGLUT1-3 and VGAT within neurons, with VGAT acting as the sole essential transporter for GABAergic inhibitory transmission. All these findings highlight the critical roles of these two neurotransmitter systems in sleep–wake regulation.

### 2.2 Mechanisms by which the GABAergic system synergistically and indirectly improves insomnia symptoms in conjunction with other systems

#### 2.2.1 Inhibition of neuroinflammation

Inflammation and apoptosis of neurons are also contributors of insomnia given their adverse effects of causing autonomic nervous system dysfunction and sleep homeostasis imbalance. Insomnia, in turn, may further trigger neuroinflammation and apoptosis following its activation of the immune system, forming a vicious cycle (Zhu et al., 2024). Circadian rhythm factors, inflammatory molecules, neurotransmitters, and physiological mechanisms that control vascular hemodynamics appear to keep a balance in the regulation of sleep homeostasis (Zielinski and Gibbons, 2022). Sleep regulation-related inflammatory molecules may have an influence in the expression of circadian clock genes, and vice versa. This may enhance or suppress sleep pressure as a whole, thereby inducing or inhibiting sleep. The sleep–wake cycle has been revealed to be mediated remarkably by glial cells, beyond neurons, and mediators of glial cells [e.g., adenosine, pro-inflammatory cytokines interleukin-1β (IL-1β), interleukin-6 (IL-6), and tumor necrosis factor-*α* (TNF-α)] may exert direct regulatory role in sleep pressure, duration, and depth (Bonilla-Jaime et al., 2021). Additionally, as evidenced by a meta-analysis (Irwin et al., 2016), patients with sleep disorders were examined with obviously escalated levels of inflammatory markers C-reactive protein (CRP) and IL-6. Furthermore, the extracellular signal-regulated kinase (ERK) pathway from the mitogen-activated protein kinase (MAPK) family can modulate cell proliferation, differentiation, apoptosis, oxidative stress (OS), immune inflammation, and other key physiological and pathological processes, thereby participating in various inflammatory diseases, mental disorders, and insomnia. Upon receiving stimulatory signals, this pathway is activated to trigger the activity of downstream transcription factor nuclear factor κB (NF-κB), inducing the release of inflammatory cytokines (e.g., IL-1β, IL-6, and TNF-*α*) and functional alterations in these cytokines. Besides causing insomnia, this change can also heighten the risk of chronic inflammatory diseases (Zhang M. M. et al., 2023). The resultant activation of p38MAPK/NF-κB pathway may further increase the secretion of inflammatory cytokines IL-1 and TNF-α to accelerate neuronal apoptosis. Blockage of p38MAPK pathway has been reported to significantly upregulate GABA_B_R expression (Li et al., 2017), underlining a pivotal regulatory role of this signaling in modulating GABA_B_R expression. GABA can inactivate NF-κB and p38 MAPK-mediated inflammatory pathways, suppressing the responsiveness of astrocytes and microglia to inflammatory stimuli (e.g., lipopolysaccharide and interferon-*γ*), thereby reducing the release of inflammatory cytokines (Lee et al., 2011). Additionally, GABA may inhibit the synergistic induction of IL-6 release by IL-1β and TNF-α (Roach et al., 2008). In summary, the GABAergic system has an established relationship with neuroinflammation, which can trigger autonomic dysfunction and sleep homeostasis imbalance to induce insomnia. Insomnia, in turn, activates the immune system and exacerbates neuroinflammation, creating a vicious cycle. Therefore, targeting the GABAergic system to improve related neuroinflammatory symptoms can be a key direction for relieving insomnia.

#### 2.2.2 Repairing oxidative damage

OS is a metabolic dysfunction resulting from the imbalance of excessive reactive oxygen species (ROS) generation and antioxidant defense capability within the body. Common ROS include superoxide anion (O_2_^−^), hydrogen peroxide (H_2_O_2_), and hydroxyl radical (OH •) (Zhao et al., 2024), where O₂^−^ is both a product of certain signaling enzymes and a byproduct of metabolic processes such as mitochondrial respiration. Superoxide dismutase can catalyze the conversion of O₂^−^ into oxygen (O₂) and H₂O₂ to modulate the level of superoxide and control levels of various ROS (Wang et al., 2018). OS has been recognized as an important factor in the pathophysiology of insomnia and may serve as a biomarker associated with insomnia (Yeung et al., 2021). ROS may accumulate within neurons during wakefulness, while sleep serves to defend against oxidative damage by clearing ROS from the brain. Conversely, insomnia stems from an imbalance between the production of ROS and the clearance capacity of the endogenous antioxidant defense system (Bin Heyat et al., 2022). There may be oxidative damage with the worsening of the level of OS (van der Pol et al., 2019). ROS is a type of neuromodulator that can activate calcium/calmodulin-dependent protein kinase II in spinal glutamatergic neurons, with a role in disinhibition by inhibiting GABAergic interneurons as well (Li J. et al., 2021). OS has been proven to cause damage to GABAergic interneurons (Scheuer et al., 2022). Therefore, one of the primary objectives in insomnia treatment is to improve OS status, thereby preventing oxidative damage or its exacerbation and exerting a protective effect on the GABAergic system.

#### 2.2.3 Enhanced neurotrophic support

Brain-derived neurotrophic factor (BDNF) works critically in regulating synaptic transmission and long-term potentiation (LTP) within the hippocampus and other brain regions (Leal et al., 2014), with a central role in neuronal and synaptic plasticity (Colucci-D'Amato et al., 2020). Insufficient sleep may decrease the level of BDNF, which may be associated with reduced BDNF mRNA expression in peripheral blood mononuclear cells and may correspond to lowered BDNF levels in the brain (Schmitt et al., 2016). Meanwhile, mild sleep deprivation can activate the cerebral cortex and brainstem, generating physiological drives for non-REM and REM sleep, respectively, accompanied by BDNF upregulation in these regions (Rahmani et al., 2020). BDNF has been reported to prolong non-REM sleep duration in rats and rabbits (Kushikata et al., 1999), suggesting its participation in sleep regulation. It is also one of the core regulatory factors for GABAergic synapses, and sustained extracellular supply of BDNF is crucial for the proper formation and functional maturation of glutamatergic and GABAergic synapses (Gottmann et al., 2009). Moreover, in the cerebral cortex and hippocampus, BDNF is synthesized and secreted in an activity-dependent manner by pyramidal neurons—the target cells of GABAergic neurons (Marty et al., 1997). In prefrontal cortex pyramidal neurons, reduced BDNF levels can reduce the activity of autophagy, resulting in p62 protein accumulation, in turn lowering the expression of α5-GABA_A_R on the cell membrane surface, thereby impairing GABAergic signaling (Tomoda et al., 2022). The activation of Tropomyosin receptor kinase B (TrkB) has also been noticed to enable the enhancement of GABA_A_R responsiveness in Purkinje neurons (Arenas et al., 2022), likely attributable to the increased phosphorylation levels and/or surface expression of GABA_A_Rs. Elevated BDNF levels can enhance TrkB activation in Purkinje neurons, resulting in the improvement of GAD65 and GAD67 levels, GABA level, membrane expression of GABA_A_R γ2, α2, and β3 subunits, as well as other outcomes that promote GABAergic system expression. In view of the above, positive regulation of BDNF can promote positive expression of the GABAergic system.

#### 2.2.4 Circadian rhythm regulation

The suprachiasmatic nucleus (SCN) of the mammalian hypothalamus, with regulatory effects on the diurnal rhythms of behavior and physiology, is considered the master circadian pacemaker (Aton et al., 2006). An autonomous transcriptional-translational negative feedback loop has been recognized to be the core of the mammalian circadian clock, with the central driver being the heterodimer formed by transcription factors CLOCK and BMAL1 (CLOCK: BMAL1). This heterodimer can bind to the E-box elements in the regulatory regions of target genes, resulting in the activated expression of Period (Per1, Per2, and Per3), Cryptochrome (CRY1, and CRY2), nuclear receptor REV-ERB (Nr1d1, and Nr1d2), etc. (Pan et al., 2020; Brzezinski et al., 2021; Koch et al., 2022; Mohawk et al., 2012). Subsequently, the accumulated PER and CRY proteins form complexes, translocate into the cell nucleus, and inhibit the transcription of CLOCK: BMAL1, forming the primary negative feedback loop. Meanwhile, the activated REV-ERB proteins can bind to the ROR response element in the Bmal1 gene promoter region, inhibiting its transcription, forming a secondary inhibitory loop. Consequently, these negative feedback mechanisms lead to periodical decline of the levels of related transcripts and proteins after accumulation. A new round of CLOCK: BMAL1 activation begins following the relief of the inhibitory effect, forming an approximately 24-h oscillation cycle (Mohawk et al., 2012; Peng F. et al., 2022). Furthermore, the secretion of melatonin (MT) is also regulated by the SCN, primarily through binding to the MT1 receptor in the SCN, thereby participating in the regulation of the CNS and circadian rhythms (Jia et al., 2024). Almost all SCN neurons express GABA, and the SCN contains VGAT, GABA_A_R, and GABA_B_R, with high expression of GAD (Ono et al., 2018). The ratio of glial cells to neurons is 1:3 in the SCN, in other word, each glial cell averages four neuronal cell bodies. Astrocytes expressing GAT-1 and GAT-3 surround synapses, neuronal cell bodies, and processes, limiting the efflux and diffusion of GABA, thereby regulating local extracellular GABA concentrations around neurons (Moldavan et al., 2015). In relative to GAT-1, GAT-3 has a higher affinity for GABA, which may be the primary transporter that modulate daytime GABA uptake to control extracellular GABA concentration in the SCN and maintain circadian rhythm balance (Patton et al., 2023). GAT-1 and GAT-3 are complementary to each other functionally, jointly regulating extracellular GABA concentrations in the SCN as well as GABA_A_R-mediated synaptic currents and tonic currents. The absence of Bmal1 in astrocytes may reduce GABA uptake and disrupt circadian rhythmic activity, an effect that can be rescued by GABA_A_R antagonists (Moldavan et al., 2017). Collectively, the circadian clock system is an important mechanism in the regulation of circadian rhythm, which has a close association with the GABAergic system.

#### 2.2.5 Steady-state modulation of the HPA axis

The HPA axis is the core neuroendocrine pathway through which the body responds to environmental stress. Stress signals can promote the release of corticotropin-releasing hormone (CRH) and arginine vasopressin (AVP) by triggering the paraventricular nucleus of the hypothalamus. These hormones act on the anterior pituitary gland, promoting the secretion of adrenocorticotropic hormone (ACTH). ACTH then stimulates the adrenal cortex to synthesize and release glucocorticoids, such as cortisol (Menke, 2024; Knezevic et al., 2023). Cortisol exerts a wide range of physiological effects by binding to the glucocorticoid receptor. Critically, cortisol works to suppress subsequent production of CRH from the hypothalamus and ACTH from the pituitary gland, through a negative feedback mechanism, thereby restoring the internal homeostasis (Menke, 2024). GABAergic neurons can suppress pituitary ACTH secretion through inhibiting the release of CRH and AVP in the hypothalamus. In non-stressful states, GABA_A_Rs with *δ* subunits on CRH neurons continuously generate inhibitory currents, effectively suppressing CRH neuron activity. Additionally, alprazolam, a GABA_A_R agonist, can strongly inhibit excessive activation of the HPA axis in both basal and stress states (Giordano et al., 2006; Camille Melón and Maguire, 2016). Insomnia can activate the HPA axis excessively to impair the regulatory function cortisol, which has more severe consequences in adolescents and may escalate the possibility of developing depression. HPA axis dysfunction is a frequently-occurring biological basis for depression and insomnia (Asarnow, 2020). With respect to the aforementioned findings, the GABAergic system is associated with the homeostasis of the HPA axis, and excessive activation of the HPA axis may increase the risk of depression and insomnia.

## 3 TCM targeting the GABA system to treat insomnia

Oral Chinese herbal medicine, acupuncture, auricular therapy, and dietary therapy are common therapies in the field of TCM that may be efficient in managing insomnia. Among these, oral Chinese herbal medicine stands out as a classic therapeutic option. It has been accepted to be the most common method in TCM for treating patients with insomnia, demonstrating significant therapeutic effects with minimal adverse reactions (He et al., 2019; Guo et al., 2024). In the following discussion, we will explore the treatment and regulation of insomnia through oral Chinese herbal medicine targeting the GABAergic system.

### 3.1 Chinese herbal formulas

In TCM, a prescription is defined as a specific herbal formula composed of at least two types of medicinal herbs. Specific herbs work in synergy to enhance therapeutic effects and reduce toxic effects. Taking such a formula orally as a decoction is one of the most classic therapeutic methods in TCM (Table 1).

**Table 1:** Therapeutic mechanisms of Chinese herbal formulas in treating insomnia based on the GABAergic system and related systems.

Chinese herbal medicine formulas	Composition	Dose	Experiment Model	Relevant indicators	Effect	Ref.
Huanglian Wendan Tang	*Coptis chinensis, Phyllostachys nigra, Citrus aurantium, Pinellia ternata, Citrus reticulata, Glycyrrhiza uralensis, Poria cocos*	0.25 mg/mL or 3 mg/mL	HEK293T cell model	Activation of α1β3γ2L GABA_A_R	Inhibiting neural excitation	(Li et al., 2024)
Huanglian Wendan Tang	Ethyl glucoside	100 μM	Wild-type AB strain zebrafish, Zebrafish larvae on day 5 post-fertilization	Activation of GABA_A_R	Inhibiting neural excitation; the excessive motor activity in the experimental model was alleviated	(Li et al., 2024)
Si Ni San	*Bupleurum chinense, Paeonia lactiflora, Citrus aurantium, Glycyrrhiza uralensis*	21.7 g/kg	Neurons in the cortex of newborn SD rats	Directly acts on GABA_A_R, Cl^−^ influx ↑	Inhibiting neural excitation	(Liu et al., 2013)
An Mie Dan	*Ziziphus jujuba* var*. Spinosa* (Crudum), *Ziziphus jujuba* var*. Spinosa* (Tostum), *Poria cum Radix Pini, Angelica sinensis, Panax ginseng, Ophiopogon japonicus, Salvia miltiorrhiza, Schisandra chinensis, Acorus tatarinowii, Glycyrrhiza uralensis*	18.18 g/kg	SPF-grade male SD rats with sleep deprivation induced by multi-platform water environment	GABA↑, Glu↓, GLT-1↑, Relative expression levels of GAD65 and GAD67 mRNA and protein expression ↑	Inhibiting neural excitation, promotes GABA synthesis; total activity time in the experimental model decreased	(Ji et al., 2024a)
Baihe Dihuang Tang	*Lilium brownii* var*. viridulum, Rehmannia glutinosa*	4.5 g/kg	PCPA intraperitoneal injection and multi-platform water environment-induced insomnia KM mice	GABA↑, Glu↓, IL-1β, IL-6, TNF-α, NF-κB gene expression↓	Inhibiting neural excitation, inhibiting neuroinflammation; the experimental model exhibited increased distance traveled into the open arms of the elevated plus maze, prolonged duration spent in the open arms, reduced sleep latency, and extended total sleep time	(Wang Y. J. et al., 2024)
Radix Ginseng and Semen Ziziphi Spinosae drug pair	*Panax ginseng, Ziziphus jujuba* var*. spinosa*	2.16 g/kg	PCPA intraperitoneal injection-induced insomnia in SPF-grade SD rats	GABA, GAD65↑, Glu↓, Gln, GS↑, mGluR5 mRNA, NR1 mRNA, GluR1 mRNA expression↓	Inhibiting neural excitation, promotes GABA synthesis, improving the GLU/GABA-GLN cycle; the experimental model exhibited a shortened sleep latency and a significantly prolonged sleep duration	(Qiao et al., 2022)
Modified Suan Zao Ren Tang	*Ziziphus jujuba* var*. spinosa, Dendrobium officinale, Ligusticum chuanxiong, Poria cocos, Glycyrrhiza uralensis*	14.4 g/kg	PCPA intraperitoneal injection and multifactorial insomnia-induced ICR mice	5-HT, 5-HT_1A_R ↑, DA, NE ↓, OX2R expression, Orexin-A ↓, GABA concentration ↑, Glu/GABA ↓, CORT, ACTH, CRH ↓	Inhibiting neural excitation, maintaining HPA axis homeostasis, 5-HT System enhancement, weakening of the orexin system, inhibition of the NE system, inhibition of the DA system; improvements in stress and anxiety-like behaviors in experimental models	(Dong et al., 2021)
Ziyin Yangxue Anshen Decoction	*Ziziphus jujuba* var*. spinosa, Schisandra chinensis, Acanthopanax senticosus, Dimocarpus longan, Polygala tenuifolia, Poria cocos*	146 g/kg or 292 g/kg	PCPA intraperitoneal injection induces insomnia in SPF-grade KM mice	GABA↑, GABA_A_Rα1 protein expression, GABA_A_Rα1 mRNA expression↑, GAD67 protein expression↑	Inhibiting neural excitation, promotes GABA synthesis	(Sun et al., 2019)

“↑” means increase, “↓” means decrease.

Huanglian Wendan Tang (HWT) is a clinically common TCM prescription composed of 7 Chinese herbs for treating insomnia with syndrome of internal disturbance of phlegm-heat. Modern pharmacological research has found that HWT may exert its anti-insomnia effects by regulating IL-6, cyclin D1, vascular endothelial growth factor A, intercellular adhesion molecule-1, and oligofructose levels, thereby modulating nuclear receptor activity pathways, neurotransmitter binding pathways, and NF-κB signaling pathways, among others (Shi et al., 2023). HWT freeze-dried powder has been proven to enable the inducement of inward currents in α1β3γ2L GABA_A_Rs, even in the absence of GABA (Li et al., 2024). These currents are sensitive to the GABA_A_R antagonist bicuculline, further indicating that the currents are mediated by GABA_A_Rs. HWT exerts GABAergic agonist effects. The active components in HWT—β-caryophyllene, (+)-cuparene, and ethyl glucoside—dose-dependently enhance GABA-induced currents, and all act as positive allosteric modulators of α1β3γ2L GABA_A_Rs. A clinical trial demonstrated that HWT significantly increased serum GABA level and alleviated adverse symptoms in insomnia patients (Cai et al., 2021).

Si Ni San is a classic formula in TCM composed of *Bupleurum chinense*, *Paeonia lactiflora*, *Citrus aurantium*, and *Glycyrrhiza uralensis*, four herbs totally. A prior research revealed that the GABA concentration-response curve of the neurons shifted to the left, under GABA stimulation, with a trend toward a decrease in EC50, when rat serum containing the Si Ni San was co-incubated with primary cultured rat cortical neurons for 3 h (Liu et al., 2013). Moreover, there was a significant increase in the maximum peak of GABA_A_R-mediated Cl^−^ current, with retarded process of current decay. Accordingly, Si Ni San can directly act on GABA_A_R in cortical neurons, enhancing the Cl^−^ influx mediated by GABA_A_R, thereby strengthening the inhibitory effect of neurons to improve insomnia.

An Mie Dan is a TCM formula composed of ten distinct herbs. Within the framework of TCM theory, it primarily targets symptoms such as palpitations, insomnia that stems from a pattern characterized by hear-Qi deficiency, and blood failing to tonify the heart (Ji et al., 2024b). Animal experiment-based research has confirmed (Ji et al., 2024a) that An Mie Dan can reduce spontaneous activity in sleep-deprived rats, increase GABA concentration in the cortex, decrease Glu concentration, and increase the relative mRNA and protein expression of GLT-1, GAD65, and GAD67, thereby improving the circadian rhythm of sleep-deprived rats. A clinical study involving 480 patients identified that An Mie Dan was effective in treating chronic insomnia in adults, with favorable safety as well (Ji et al., 2024b).

Baihe Dihuang Tang (BDT) is a well-known herbal formula in TCM composed of Lilium and raw rehmannia, commonly adopted for treating depression, insomnia and other mental disorders extensively, has a positive effect on the treatment and prognosis of insomnia patients (Peng L. et al., 2022). Research on its active components has revealed neuroactive properties possessed by saponins, cycloartenol glycosides, and polysaccharides. Catalpol, a cycloartenol glycoside abundant in Rehmannia, has been found to exhibit neuroprotective antioxidant capability and shows potential in relieving inflammation-related neurodegenerative diseases (Chen et al., 2012). In a study with the construction of an insomnia mouse model via para-Chlorophenylalanine (PCPA) intraperitoneal injection combined with multi-factor stimulation, BDT increased GABA concentration and decreased Glu concentration in the brain of insomniac mice; downregulated the expression levels of IL-1β, IL-6, TNF-*α*, and NF-κB genes in colon tissue, significantly shortened the sleep latency, and increased the sleep duration of insomniac mice (Wang Y. J. et al., 2024). Hence, BDT can effectively treat symptoms related to insomnia, which may be attributed to the regulation of neurotransmitter disorders in the brain combined with the relieving-effect of inflammatory responses.

The combination of two specific herbs is also known as an herbal pair. Ginseng has numerous benefits, such as calming the mind, enhancing intelligence, tonifying the spleen and lungs, as well as nourishing Qi and blood (Liu et al., 2020). *Ziziphus jujuba* seeds are primarily used in TCM to treat insomnia (Ruan et al., 2024). Simultaneously, mGluR5 mRNA, with a wide distribution in the brain, is associated with various neurological disorders. The transcription level of NR1 mRNA in the ventrolateral preoptic area (VLPO) nucleus region is positively correlated with sleep stress; moreover, complete sleep deprivation can trigger a sharp increase in the expression of GluR1 mRNA in the cortex and hippocampus regions. It has been pointed out that the ultrafiltrate of Radix Ginseng and Semen Ziziphi Spinosae drug pair (R-S) can contribute to the increase of GABA and GAD65 levels in the hippocampus of insomniac rats, reduction of Glu level, enhanced neural inhibition while reduced excitability, increase of Gln and GS levels, and decrease of mGluR5, NR1, and GluR1 mRNA expression levels (Qiao et al., 2022). Moreover, it also improved the intestinal microcirculation of insomniac rats, confirming its effect in relieving insomnia symptoms by regulating the intestinal microbiota structure and Glu/GABA-Gln metabolic cycle in rats.

Suan Zao Ren Tang (SZRT), with the effects of nourishing blood and calming the mind, is a classic formula in TCM for treating insomnia. As a small-molecule indoleamine, 5-Hydroxytryptamine (5-HT) has been validated to be involved in various physiological functions such as memory, cognition, and the sleep–wake cycle. It is one of the earliest neurotransmitters identified to be related to the physiological modulation of the proposed cycle (Kantor et al., 2004; Charnay and Léger, 2010). The 5-HT_1A_ receptor (5-HT_1A_R) is involved in the spontaneous and homeostatic regulation of REM sleep (Boutrel et al., 2002). Ocytocin neurons are a key component of the “awake maintenance system” (Ferrari et al., 2018). Selective blockade of orexin receptor 2 (OX2R) can promote sleep (Dugovic et al., 2009). Dopamine (DA) is an important regulatory factor for neurons and synapses in the CNS, and the DA D2 receptor (D2R) is a crucial receptor for maintaining wakefulness (Cao et al., 2022). Moreover, norepinephrine (NE) is one of the primary neurotransmitters involved in wakefulness (Mitchell and Weinshenker, 2010). Modified SZRT (MSZRD) has been reported to escalate hypothalamic protein levels of 5-HT and 5-HT_1A_R in insomniac mice, downregulate DA and NE levels, reduce the expression of OX2R; increase serum GABA concentration and decrease Orexin-A levels; and also downregulate levels of HPA axis-related hormones corticosterone (CORT), ACTH, and CRH. In addition, MSZRD has been discovered to enable a joint regulation of the 5-HT system, GABA system, HPA axis, and orexin system, thereby achieving the alleviation of symptoms associated with circadian rhythm disorders, shorten sleep latency, and prolong sleep duration (Dong et al., 2021).

Ziyin Yangxue Anshen Decoction (ZYAD) consists of six herbs (e.g., *Ziziphus jujuba* seeds and Schisandra chinensis). JuA, the primary active component of *Ziziphus jujuba* seeds, can enhance the gene transcription level of GABA receptors in neurons, beyond a significant inhibition of the excitatory signaling pathway mediated by Glu in neurons, thereby achieving sedative and hypnotic effects (Wang et al., 2015). Schisandrin B (SchB) is an active lignan component derived from Schisandra chinensis. Moreover, its sleep-promoting effect is closely related to the GABAergic system (Li et al., 2018). As evidenced by related animal experiments (Sun et al., 2019), ZYAD can improve sleep in insomniac mice by increasing GABA level, enhancing hypothalamic GABA_A_Rα1 protein expression and GABA_A_Rα1 mRNA expression, as well as increasing hypothalamic GAD67 protein expression.

### 3.2 Chinese patent medicines

Chinese patent medicines is made from Chinese herbal medicines, which are processed according to standards into convenient clinical preparations, including tablets, pills, oral liquids, capsules, and other dosage forms (Table 2).

**Table 2:** Therapeutic mechanisms of Chinese patent medicines in treating insomnia based on the GABAergic system and related systems.

Chinese patent medicines	Composition	Dose	Experiment Model	Relevant indicators	Effect	Ref.
Jiu Wei Bu Xue Oral Liquid	*Gynostemma pentaphyllum, Panax ginseng, Astragalus membranaceus, Schisandra chinensis, Angelica sinensis, Atractylodes macrocephala, Citrus reticulata, Crataegus pinnatifida, Glycyrrhiza uralensis*	8 mL/kg	PCPA intraperitoneal injection-induced insomnia in SPF-grade SD rats	GAD67 activity and expression ↑, GABA_A_Rα1 and GABA_A_Rβ2 expression ↑, GABA ↑, Glu ↓, Glu/GABA ratio ↓	Inhibiting neural excitation, promotes GABA synthesis; the experimental model exhibited a shortened sleep latency, prolonged sleep duration	(Wei et al., 2023)
Compound Chaijin Jieyu tablets	*Bupleurum chinense, Curcuma wenyujin, Hypericum perforatum, Curcuma longa, Paeonia lactiflora, Anemarrhena asphodeloides, Poria cocos, Polygala tenuifolia, Acorus tatarinowii, Panax ginseng*	5.4 g/kg or 10.8 g/kg	CUMS combined with chronic sleep deprivation induces insomnia in SPF-grade SD rats	Glu↓, GABA↑, GAD67, GABA_A_R, GABA_B_R protein and gene expression↑	Inhibiting neural excitation, promotes GABA synthesis; the experimental model exhibited a shortened sleep latency, prolonged sleep duration, increased sleep onset rate	(Liu et al., 2021)
Naoxinshu Oral Solution	Royal Jelly, Armillaria mellea Fermented Concentrate	2.60 mL/kg	SPF-grade male ICR mice	GABA↑, Glu↓, GABA-T activity and expression↓, GAD enzyme activity and expression↑	Inhibiting neural excitation, promotes GABA synthesis, inhibition of GABA degradation; the experimental model exhibited a shortened sleep latency, prolonged sleep duration, increased sleep onset rate, decreased frequency of spontaneous activity	(Qi et al., 2024)
Shenmu Anshen Capsules	*Ziziphus jujuba* var*. spinosa* (Tostum)*, Platycladus orientalis, Polygala tenuifolia, Cyperus rotundus, Rehmannia glutinosa, Lycium barbarum, Schisandra chinensis, Borneolum Syntheticum, Magnetitum Calcination, Os Draconis, Concha Ostreae, Crataegus pinnatifida,* Chao Liu Shen Qu (Stir-fried Massa Fermentata)	3.69 g/kg	PCPA intraperitoneal injection induces insomnia in SPF-grade C57 mice	DA, cortisol↓, GABA, MT, BDNF↑, GABA_A_Rβ3, PKA, CREB protein expression↑	Inhibiting neural excitation, circadian rhythm regulation, enhance neurological support, inhibition of the DA system; the experimental model exhibited shorter wakefulness duration and longer NREM and REM sleep periods, resulting in a more complete sleep architecture	(You et al., 2025)

“↑” means increase, “↓” means decrease.

Jiu Wei Bu Xue Oral Liquid (JWBXOL) is formulated with *Gynostemma pentaphyllum*, a unique herb from Guangxi, combined with ginseng, astragalus, and seven other traditional Chinese herbs. With functions of tonifying Qi and improving the blood circulation, as well as strengthening the spleen and stomach, this formula is particularly effective in treating insomnia, forgetfulness, and palpitations caused by Qi-blood deficiency. In an intraperitoneal PCPA injection-induced insomniac rat model, JWBXOL was found to enhance GABA synthesis by improving the activity and expression of GAD67 in the hippocampal tissue (Wei et al., 2023). It can also upregulate the expression of GABA_A_Rα1, GABA_A_Rβ2 to enhance the inhibitory function of the GABAergic nervous system. Besides, it can reduce hypothalamic Glu level, lower the Glu/GABA ratio in the brain, and restore the excitatory-inhibitory balance, thereby exerting its therapeutic effects on insomnia.

Compound Chaijin Jieyu tablets (CCJT), formulated by 10 Chinese herbal ingredients, are primarily used for mild to moderate depression accompanied by insomnia caused by liver-Qi stagnation, spleen deficiency, and deficiency of the heart and mind. CCJT can reduce hippocampal and hypothalamic Glu level sharply, while increasing GABA level, in insomniac rats induced by depression (Liu et al., 2021). Mechanistically, CCJT can effectively reverse the downregulation of GAD67, as well as GABA_A_R and GABA_B_R protein and gene expression in insomniac rats, thereby restoring GABA synthesis and its normal binding function with receptors, ultimately alleviating symptoms in depressive insomnia (Liu et al., 2021).

Naoxinshu Oral Solution (NOS) is composed of royal jelly and fermented concentrated extract of *Armillaria mellea*. Both the water extract and ethanol extract of *Armillaria mellea* fermented liquid can prolong the REM sleep and non-REM sleep cycles in rats, with detectable GABA in the water extract (Li I. C. et al., 2021). Meanwhile, NOS can increase GABA levels in mouse prefrontal cortex and hippocampus, reduce Glu level, decrease GABA-T activity and expression in mouse brain tissue, while increasing GAD enzyme activity and expression (Qi et al., 2024). Thus, it can improve the sleep onset rate, shorten sleep latency, and prolonged the sleep duration in mice.

Shenmu Anshen Capsules (SAC) are composed of 13 Chinese herbal ingredients that are suitable for indications of nervous exhaustion, insomnia with frequent dreams, and heart-Yin deficiency-induced palpitations. Chronic insomnia may weaken the signal transduction of cyclic adenosine monophosphate (cAMP), a pathway that exerts its effects by activating protein kinase A (PKA), which consists of a regulatory subunit (PKAr) and a catalytic subunit (PKAc). In the context of PKAc dissociating from PKAr, activated PKA can phosphorylate downstream cAMP response element-binding protein (CREB) and exert its transcription factor function. Damage to the cAMP/PKA pathway can decrease the activity of phosphorylated CREB to affect neuronal excitability and its mediation of BDNF expression (Wang Z. et al., 2024; You et al., 2025). SAC has been proven experimentally to downregulate DA and cortisol levels in the brain tissue of insomniac mice, while upregulating GABA, MT, and BDNF levels (You et al., 2025). It would further increase GABA_A_Rβ3, PKA, and CREB protein expression levels in brain tissue, indicating that SAC can activate the cAMP/PKA/CREB pathway to facilitate the inhibition of neuronal hyperexcitability and exert sleep-promoting effect. Mechanistically, cortisol, MT, GABA, DA, and BDNF can interact synergistically to be involved in the modulation of this sleep related cycle, ultimately improving mouse sleep quality, and extending sleep duration.

### 3.3 Compounds and extracts from Chinese herbal medicines

TCM is renowned for its macro-level approach to regulate physical functions. As researchers delve deeper into the study of TCM, great concern has been attached to analyses on the specific effects of individual herbs, resulting in the clarification of the active ingredients of various Chinese herbs increasingly (Table 3).

**Table 3:** Therapeutic mechanisms of Chinese herbal medicines in treating insomnia based on the GABAergic system and related systems.

Chinese herbal medicines	Compounds or extracts	Dose	Experiment model	Relevant indicators	Effect	Ref.
*Ziziphus jujuba* seeds	Jujuboside A	18 mg/kg	PCPA intraperitoneal injection-induced insomnia in SPF-grade C57BL/6 mice	GABA↑, Glu↓, GABA_A_R_,_ GABA_B_R expression↑, NMDA and AMPA receptor expression↓	Inhibiting neural excitation; the sleep rate of the experimental model significantly increased, the sleep latency significantly shortened, and the sleep duration significantly prolonged	(Wang et al., 2025)
*Ziziphus jujuba* seeds	Jujuboside B, jujubogenin	50 μg/mL	SD rat hippocampal neurons	GABA_A_Rα1 and GABA_A_Rα5 expression ↑, GABA_A_R chloride channel opening frequency ↑	Inhibiting neural excitation	(Song et al., 2017)
Cnidii Fructus	Total Coumarins from *Cnidium monnieri*, Osthole, Imperatorin, Isopimpinellin, Bergapten	100 mg/kg or 100 mg/kg	Hippocampal tissue of CL-grade SD rats with PCPA intraperitoneal injection-induced insomnia	Clock and Bmal1 gene expression ↓, Cry1, Per1, and Per2 gene expression ↑, Glu expression ↓, GABA expression ↑	Inhibiting neural excitation, circadian rhythm regulation	(Wei et al., 2018)
Schisandra chinensis	Schisandrin B	3.5 mg/kg	PCPA intraperitoneal injection induces insomnia in Wistar rats	5-HT, 5-HIAA ↑, 5-HT_1A_R ↑, GABA expression ↑, Glu expression ↓, GAD activity ↑, GABA-T activity ↓, GABA_A_Rα1, GABA_A_Rγ2 protein expression ↑	Inhibiting neural excitation, promotes GABA synthesis, inhibition of GABA degradation, 5-HT System enhancement; the experimental model exhibited a significantly shortened sleep latency and markedly prolonged sleep duration	(Wang et al., 2020)
Radix Polygalae	Tenuifolin	40 mg/kg or 80 mg/kg	CL-grade ICR mice	GABA↑, NE↓	Inhibiting neural excitation, inhibition of the NE system; the experimental model significantly prolonged total sleep time by increasing the duration of both NREM and REM sleep	(Cao et al., 2016)
Ginseng	Ginsenoside Rg5/Rk1	60 mg/kg	Male KM mice	GABA 、5-HT ↑	Inhibiting neural excitation, 5-HT System enhancement; the experimental model significantly reduced activity levels, shortened sleep latency, and prolonged sleep duration	(Shao et al., 2020)
Ginseng	Ginsenoside Rg5/Rk1	60 mg/kg	Brain tissue from Male Wistar rats	GABA↑, Glu↓, GABA/Glu↑, GABA_A_R, GABA_B_R expression↑, 5-HT_1A_R expression↑	Inhibiting neural excitation, 5-HT System enhancement	(Shao et al., 2020)
Lilium	*Lilium davidii* bulb acetone extract	370.44 mg/kg	PCPA intraperitoneal injection-induced insomnia in SPF-grade Wistar rats	CRH、ACTH、CORT↓, 5-HT、MT↑, NE↓, 5-HT_1A_R 、 GABA_A_R ↑	Inhibiting neural excitation, maintaining HPA axis homeostasis, circadian rhythm regulation, 5-HT System enhancement, inhibition of the NE system; abnormal excitability in the experimental model was alleviated	(Si Y. et al., 2020)
Lilium	*Lilium brownii* bulb ethanol extract	598.64 mg/kg	PCPA intraperitoneal injection-induced insomnia in SPF-grade Wistar rats	5-HT、MT↑, NE↓, 5-HT_1A_R、 GABA_A_R ↑	Inhibiting neural excitation, circadian rhythm regulation, 5-HT System enhancement, inhibition of the NE system; the experimental model exhibited weight gain and increased gut microbiota diversity; histopathological examination revealed well-organized cellular arrangement and a marked reduction in the number of atrophic cells	(Si et al., 2022)
*Glycyrrhiza glabra*	Gabridin	30 μM	Expression of GABAA receptors in *Xenopus laevis* oocytes	GABA activates receptors that generate currents↑, GABA efficacy↑	Inhibiting neural excitation	(Hoffmann et al., 2016)
*Glycyrrhiza glabra*	*Glycyrrhiza glabra* ethanol extract	500 mg/kg	ICR mice and male C57BL/6 N mice	Positive allosteric modulation of GABAA-BZD receptors	Inhibiting neural excitation; the experimental model exhibited a shortened sleep latency, prolonged sleep duration, and increased non-REM sleep duration	(Cho et al., 2012)
*Glycyrrhiza glabra*	Glabrol	50 mg/kg	The cerebral cortex of SD rats	Positive allosteric modulation of GABAA-BZD receptors	Inhibiting neural excitation; the experimental model exhibited prolonged sleep latency and reduced sleep duration	(Cho et al., 2012)
fu-ling	Pachymic Acid	10 μM or 50 μM	Primary cultured neurons from the hypothalamus of SD rats	Cl^−^ influx ↑; GAD65/67 ↑	Inhibiting neural excitation, promotes GABA synthesis	(Shah et al., 2014)

“↑” means increase, “↓” means decrease.

*Ziziphus jujuba* seeds are the dried mature seeds of the plant *Ziziphus jujuba* Mill. var. *spinosa* (Bunge) Hu ex H. F. Chou of the Rhamnaceous family. As a TCM herb, *Ziziphus jujuba* seeds are a common option for treating insomnia. As a triterpenoid saponin isolated from *Ziziphus jujuba* seeds, Jujuboside A (JuA) has been shown to possess antioxidant, anti-inflammatory, anti-apoptotic, and neuroprotective effects, as well as various bioactive properties (Zhong et al., 2024). AMPAR and NMDAR are the primary ionotropic Glu receptors, with AMPARs mediating the majority of fast excitatory synaptic transmission (Jin et al., 2023). Parvalbumin-positive/Somatostatin-positive (PV+/SST+) interneurons constitute the major component of GABAergic interneurons (Folorunso et al., 2023). JuA can enhance PV+/SST + neuron activity, increase GABA level, reduce Glu content, upregulate GABA_A_R and GABA_B_R protein expression, and downregulate NMDA and AMPA receptor expression (Wang et al., 2025). Pharmacological inhibition of the GABA signaling can eliminate the therapeutic effect of JuA, further validating its insomnia-relieving effect by acting on the GABAergic system. Jujuboside B (JuB) and jujubogenin are the primary metabolites of JuA. In a rat hippocampal neuronal model, JuB and jujubogenin enabled the upregulation of GABA_A_Rα1 and GABA_A_Rα5 expression levels (Song et al., 2017). The GABA_A_Rα1 subunit primarily mediates sedative-hypnotic effects, while the GABA_A_Rα5 subunit modulates muscle relaxation and accelerates the onset of sedative-hypnotic effects. In the study using MQAE fluorescent probe technique, researchers acquired direct evidence that JuB and jujubogenin can increase the opening frequency of GABA_A_R Cl^−^ channels, and their sedative-hypnotic effects may primarily be achieved by increasing the number of GABA_A_Rs on the surface of hippocampal neurons (Song et al., 2017).

*Cnidii Fructus* is the dried mature fruit of *Cnidium monnieri* (L.) Cuss. of the Apiaceae family, with effects of warming the kidney to invigorate Yang in TCM. Modern pharmacological research has shown that *Cnidii Fructus* and its main components have sedative and hypnotic effects to be effective in improving cerebral ischemia (Hou et al., 2015). Experiments have found (Wei et al., 2023) that the hypnotic active components of the TCM *Cnidii Fructus* (total coumarins from *Cnidium monnieri*, *Osthole*, *Imperatorin*, *Isopimpinellin*, and *Bergapten*) can reduce hippocampal expression levels of the Clock and Bmal1 genes in insomniac rats to upregulate Cry1, Per1, Per2 gene expression, downregulate Glu expression obviously, and increase GABA expression, thereby regulating the sleep–wake cycle.

Furthermore, SchB, a primary bioactive constituent from *Schisandra chinensis* (Turcz.) Baill, has been uncovered to possess multiple neuroprotective effects, sedative, and hypnotic activities. It can eliminate the inhibitory effect of pentylenetetrazole on GABA-induced currents (Wu et al., 2024). It has been proposed that SchB can improve insomnia in rats through the 5-HT and GABA systems, including increasing hypothalamic levels of 5-HT and 5-hydroxyindoleacetic acid (5-HIAA), 5-HT_1A_R content, GABA expression, GAD activity, and obviously upregulating GABA_A_Rα1 and GABA_A_Rγ2 protein expression; as well as decreasing Glu expression levels, significantly reducing GABA-T activity (Wang et al., 2020).

Radix Polygalae is a TCM whose dried root has been extensively utilized to enhance intelligence and calm the mind. Tenuifolin is a saponin extracted from Polygala root that exhibit remarkable anxiolytic and sedative-hypnotic activities. Tenuifolin may induce sleep, manifested as prolonged total sleep time, by activating the GABAergic system and inhibiting the noradrenergic system (Cao et al., 2016). Neurotransmitter analysis revealed increased GABA levels in the VLPO, locus coeruleus (LC), and perifornical area (Pef), as well as reduced NE levels in the LC, VLPO, pontomesencephalic tegmental area (PPT), and laterodorsal tegmental area (LDT).

Ginseng is the dried roots and rhizomes of *Panax ginseng* from the Araliaceae family. As unveiled by existing publication, ginsenoside Rg5 can remarkably increase GABA levels in all brain tissues and abdominal aorta blood of rats, while decreasing hippocampal, hypothalamic and serum Glu levels (except the cerebral cortex); while ginsenoside Rk1 can obviously decrease Glu levels in all brain tissues and blood, and increase GABA levels in the hypothalamus, cerebral cortex, and abdominal aorta blood (except the hippocampus) (Shao et al., 2020). Western blot analysis confirmed that Rg5/Rk1 can upregulate GABA_A_R (α1, α3, γ2, β1 subunits), GABA_B_R (1a, 1b, 2 subunits), and 5-HT_1A_R protein expression in rat brain tissue. Additionally, picrotoxin, a selective GABA_A_R antagonist, and SCH50911, a GABA_B_R antagonist, can both antagonize the sedative-hypnotic effects induced by Rg5/Rk1 in mice. In addition, Rg5 and Rk1 also functioned to escalate GABA and 5-HT levels in mouse cecum significantly, confirming their sedative and hypnotic effects by acting on the GABAergic and serotonergic systems.

Lilium, a TCM herb, has a calming effect. Acetone extracts of *Lilium davidii* can reduce serum levels of CRH, ACTH, and CORT in insomniac rats, thereby treating HPA axis hyperactivity (Si Y. et al., 2020). These researchers also found elevated hypothalamic levels of 5-HT and MT, decreased NE levels, as well as highly increased 5-HT_1A_R and GABA_A_R levels, indicating a mechanism involving the combined regulation of the GABAergic system, 5-HT System, HPA axis, and MT. In subsequent experiments on *Lilium brownii* ethanol extract (Si et al., 2022), similar changes were noticed in hypothalamic levels of neurotransmitter, hormone, and receptor expression levels in insomniac mice, further validating the accuracy of the experimental results.

*Glycyrrhiza glabra* is an extremely important herb in TCM, appearing in most prescriptions and known as “Guolao.” Glabridin, one of the main flavonoid compounds in *Glycyrrhiza glabra*, has a strong enhancing effect on heterologously expressed GABA_A_R. It can significantly enhance the current generated by GABA-activated receptors and increase the efficacy of GABA (Hoffmann et al., 2016). *Glycyrrhiza glabra* ethanol extract (GGE) and its active flavonoid compound glabrol may promote sleep through allosteric modulation of GABA_A_-BZD receptors (Cho et al., 2012). GGE can shorten sleep latency period and prolongs sleep duration, and its hypnotic effect is inhibited by flumazenil. Glabrol was tested in [^3^H]flumazenil binding experiments using receptor membrane preparations from rat cerebral cortex. TIt was proven to suppress the binding of [^3^H]flumazenil to rat cerebral cortex membranes, with key roles of the two isoprenyl groups of glabrol found in binding to GABA_A_-BZD receptors and exerting sedative-hypnotic effects.

*Poria cocos* is a saprophytic fungus whose sclerotia are known as fu-ling, acting as health supplements in TCM. Research has indicated that PA, the primary lanostane-type triterpenoid compound in *Poria cocos*, possesses sedative and hypnotic activity (Wei et al., 2022). PA treatment of hypothalamic neurons can significantly increase intracellular Cl^−^ influx and enhance GAD65/67 protein levels, indicating its potential to prolong sleep duration and reduce sleep latency by modulating the GABAergic system (Shah et al., 2014).

## 4 Conclusion

**Figure 2:**
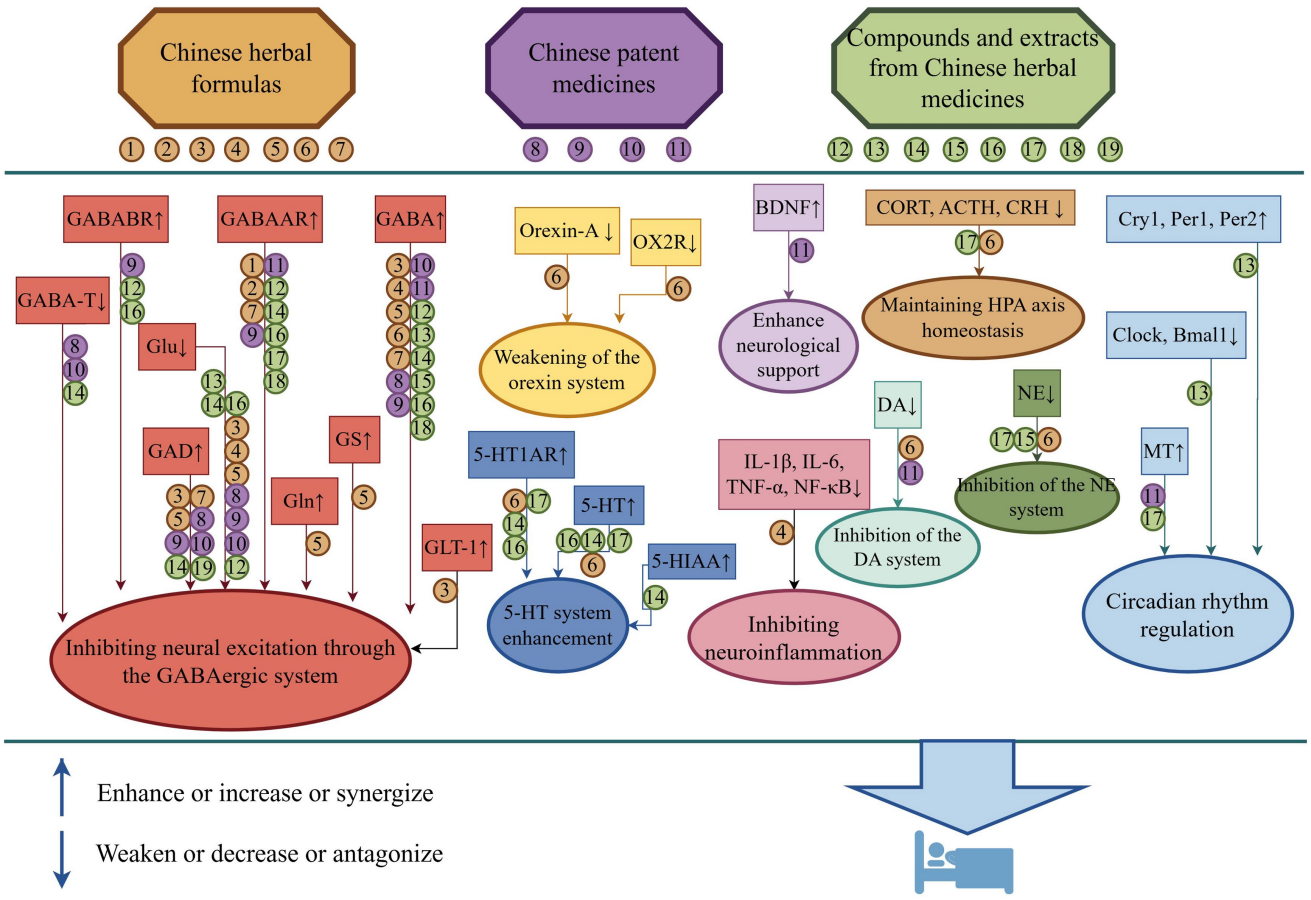
Therapeutic mechanisms of TCM in treating insomnia based on the GABAergic system and related systems. *Note:* Therapeutic mechanisms of TCM in treating insomnia based on the GABAergic system and related systems using Figdraw (www.figdraw.com). 1: Huanglian Wendan Tang; 2: Si Ni San; 3: An Mie Dan; 4: Baihe Dihuang Tang; 5: *Radix Ginseng* and *Semen Ziziphi Spinosae* drug pair; 6: Modified Suan Zao Ren Tang; 7: Ziyin Yangxue Anshen Decoction; 8: Jiu Wei Bu Xue Oral Liquid; 9: Compound Chaijin Jieyu tablets; 10: Naoxinshu Oral Solution; 11: Shenmu Anshen Capsules; 12: *Ziziphus jujuba* seeds; 13: *Cnidii Fructus*; 14: *Schisandra chinensis*; 15: *Radix Polygalae*; 16: Ginseng; 17: *Lilium*; 18: *Glycyrrhiza glabra*; 19: *fu-ling*.

According to the category of TCM, Chinese herbal medicine formulas, Chinese patent medicines, as well as Compounds and extracts from Chinese herbal medicines are confirmed with the effect of intervening in the GABAergic system. A direct activation of GABA_A_R can increase Cl^−^ influx to trigger neuronal hyperpolarization and suppress neural excitability. It can also increase GABA_A_R and GABA_B_R at the protein and gene levels, enhance GABA concentration by upregulating the mRNA and protein expression and activity of GAD65 and GAD67, and reduce GABA-T activity and expression, thereby decreasing GABA degradation. It can lower Glu concentration, potentially through the Glu/GABA-Gln cycle, by upregulating GLT-1 and GS, and promoting the conversion of Glu to Gln. Beyond a direct regulation of the GABAergic system, TCM also exert modulatory role on the 5-HT System by increasing 5-HT and its metabolite 5-HIAA levels, and significantly upregulating 5-HT_1A_R expression. It can downregulate DA and NE levels to inhibit excitability, lower Orexin-A, and its receptor OX2R expression to promote sleep, increase PKA and CREB protein expression levels, and activate the cAMP/PKA/CREB pathway, thus inhibiting neuronal overexcitability while promoting sleep. Moreover, it can inhibit neuroinflammation by reducing IL-1β, IL-6, TNF-*α*, and NF-κB gene expression, enhance neurotrophic support through increasing BDNF, and maintain HPA axis homeostasis via lowering CRH, ACTH, and CORT levels, while regulating circadian rhythms on the basis of downregulating the expression of core clock genes Clock and Bmal1, upregulating the expression of clock-controlling genes Cry1, Per1, and Per2, and increasing MT levels. Supported by these interconnected and interrelated functions, it may eventually facilitate the development of a complex functional network of the GABAergic system that improves sleep. Figure 2 elucidates the underlying mechanism of TCM in treating insomnia through the GABAergic system and related systems.

## 5 Limitations and future prospects

With absolute advantages of minimal side effects, broad applicability, and strong compatibility (Ye et al., 2024), TCM has achieved significant success in treating insomnia-related conditions in recent years. Noticeably, there is a continuous growth in the trend toward integrating traditional Chinese and Western medicine over the past century. It is possible to leverage the advantages of the macro-regulatory approach of traditional medicine and the micro-regulatory approach of modern medicine by integrating combining modern medical technology with the traditional theoretical foundations of TCM for disease diagnosis and treatment, without being confined by rigid boundaries. The GABAergic system is a key focus in the diagnosis and treatment of insomnia, providing diverse opportunities for drug development given its complex receptor subtypes and regulatory mechanisms. Both neuroscience and clinical medicine may be benefited a lot through a thorough understanding of the dynamic balance of the GABAergic system.

Given the involvement of the GABAergic system in multiple human regulatory systems, TCM can directly modulate the GABAergic system or act in conjunction with other signaling pathways to improve insomniac symptoms or indicators. It highlights the multi-component, multi-target, personalized, and low-dependency features of TCM, while also closely aligning with modern medicine. However, most studies have primarily focused on macro-level changes in GABA receptor expression or neurotransmitter levels, with insufficient analysis on the molecular mechanisms of specific signaling pathways. Furthermore, the targeting mechanisms of TCM remain unclear, given its multi-targeted and multi-component nature. Its personalized therapeutic approach stems from the syndrome differentiation and treatment principles of TCM, which sometimes yield remarkable efficacy, yet coupled with low reproducibility. The lack of clinical data on the application of TCM for the treatment of insomnia through the GABAergic system also poses a significant challenge. Meanwhile, there is a scarcity in the research on the interactive details between GABAergic regulation and other systems, necessitating extensive research to populate databases. Therefore, we should continue to get into the bottom of the specific mechanisms linking TCM with the GABAergic system, and conduct high-quality, large-scale clinical observational studies, combined with enhanced research on TCM components, and optimization of extraction techniques. Eventually, we hope to comprehensively unearth the active medicinal components, and sharpen understanding of the interactions between the GABAergic system and other signaling pathways. Collectively, TCM regulation of the GABAergic system holds clinical application potential for insomnia treatment. Future efforts should integrate modern technologies to deepen mechanism research, advance the accumulation of evidence-based medical evidence, thereby providing safer, personalized TCM-based therapeutic options for global insomnia management.
